# Comparative fermentability of a glucose/xylose enriched enzymatic slurry using different yeast strains

**DOI:** 10.1007/s00449-026-03399-3

**Published:** 2026-07-24

**Authors:** Lina M. Durán, Miguel A. D. Flores-Alarcón, Adriane M. F. Milagres, Inês C. Roberto

**Affiliations:** Department of Biotechnology, Engineering College of Lorena, University of São Paulo (USP), Estrada Municipal do Campinho, N° 100, Campinho, Lorena, SP 12602-810 Brazil

**Keywords:** Sugarcane bagasse, Pretreatment, Enzymatic slurry, Fermentation, Non-conventional yeasts, Second-generation ethanol

## Abstract

Lignocellulosic biomass represents a sustainable energy source as ethanol fuel demand increases; process integration and efficient C6/C5 sugars utilization are essential for achieving techno-economically viable production. Here, alkaline-sulfite pretreatment combined with disc refining was applied to sugarcane bagasse for conserving most of cellulose and hemicellulose fractions. Then, β-glucosidase supplementation of cellulase at rational enzyme dosage (18 FPU/g biomass) was established for the enzymatic hydrolysis of the pretreated solids, achieving approximately 75% conversion yield for both cellulose and xylan. The resulting enzymatic slurry was directly fermented without solid-liquid separation and used to evaluate the effects of temperature and nutrient supplementation on ethanol fermentation by three yeast strains. Under optimal conditions (30 °C with nutrients), *Kluyveromyces marxianus* Y-6860 exhibited higher ethanol productivity (${Q_P}$ = 3.25 g L^− 1^ h^− 1^) than *Saccharomyces cerevisiae* PE-2 (${Q_P}$ = 2.17 g L^− 1^ h^− 1^). Notably, *Scheffersomyces stipitis* Y-7124 was the only strain capable of significantly consuming xylose, resulting in 46% higher ethanol production than the other strains. To simulate industrially relevant conditions, the slurry was supplemented with anhydrous glucose up to 100.0 g L^− 1^. Under these conditions, *K. marxianus* Y-6860 and *S. cerevisiae* PE-2 maintained robust fermentation performance, while *S. stipitis* Y-7124 exhibited limited xylose uptake during the evaluated period. The results demonstrate the potential of direct slurry fermentation for integrated 2G ethanol production, with *K. marxianus* Y-6860 showing strong potential for industrial-scale glucose fermentation and *S. stipitis* Y-7124 being a prospective candidate for improving xylose conversion.

**Graphical abstract:**

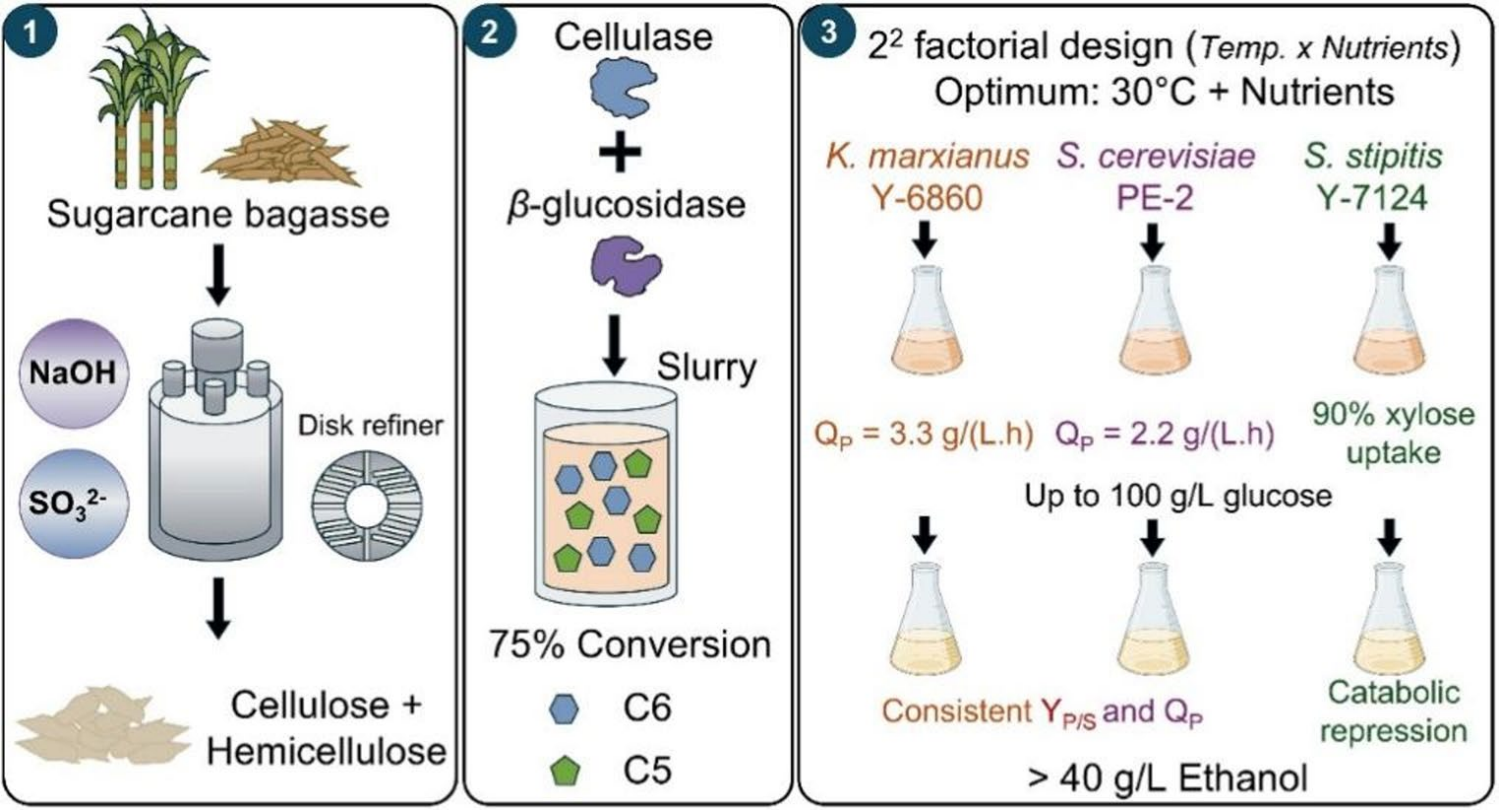

**Supplementary Information:**

The online version contains supplementary material available at 10.1007/s00449-026-03399-3.

## Introduction

Ethanol is currently one of the most widely produced renewable fuels in the world, and its demand is expected to increase as consumption patterns evolve, driven by growing demand for energy, concerns about dependence on fossil fuels, geopolitical dynamics, and the search for economic diversification [1]. In 2025, the leading producers of this biofuel were the United States and Brazil, accounting for 52 and 27%, respectively, of global production of 32.0 billion gallons [2]. In the United States, first-generation (1G) ethanol production is corn-based. In Brazil, although corn ethanol production presents excellent growth perspectives, sugarcane is still the primary feedstock [3, 4].

During the processing of sugarcane in industries, this feedstock is subjected to an extraction (milling) process to obtain a sucrose-rich juice. When the juice is evaporated and crystallized, sucrose (sugar) is obtained for food applications. On the other hand, it is transformed into ethanol when fermented and distilled – principally for the fuel market [5]. Industries generally produce a mix of sucrose and ethanol to participate in both market segments [6]. However, during the sugarcane milling process, a fibrous byproduct known as bagasse is generated.

Sugarcane bagasse is the most widely available byproduct in sugarcane-based ethanol production plants. In Brazil, for example, the production of sugarcane bagasse is estimated in approximately 186 million tons per year, considering that 666.4 million tons of sugarcane were produced in the period 2025/2026 [7] and 280 kg of bagasse are generated per ton of processed sugarcane at approximately 50% moisture. This byproduct can be burned to produce energy through cogeneration, contributing to the sustainability of ethanol production plants and, in some cases, sell surplus electricity [8].

Alternatively, as a lignocellulosic biomass, sugarcane bagasse can undergo further processing to produce second-generation (2G) ethanol without the need to expand cultivation areas. This approach can reduce the environmental impacts associated with land use and diversify the energy matrix, while helping to meet the growing demand for biofuels [9]. Furthermore, existing industrial infrastructure, combined with the on-site availability of feedstock, enables the integration of 1G and 2G ethanol production, with residual lignin also serving as a source for cogeneration [10]. In fact, considerable efforts have been made to promote and consolidate the industrial production of 2G ethanol in Brazil. However, some factors involved in the processing of lignocellulosic biomass, such as the high import and logistic costs of chemicals – for pretreatment and enzymatic cocktails – and the incomplete utilization of all carbohydrate fractions (cellulose and hemicellulose), compromise the economic sustainability of 2G ethanol plants [11].

For the conversion of lignocellulosic biomass into 2G ethanol, pretreatment is the technical step necessary to overcome the natural recalcitrance of feedstock and improve saccharification. One possible way to achieve this is through a chemical-thermomechanical (CTM) process, such as alkaline-sulfite pretreatment combined with disc refining [12]. In addition to the advantage of employing mature technology from the pulp and paper industry, the alkaline-sulfite-CTM process – applied as a pretreatment – produces physicochemical changes in lignocellulosic biomass, increasing fiber swelling and water retention capacity, thereby improving the enzymatic hydrolysis performance of carbohydrates [12, 13].

Due to the presence of a hemicellulose type structurally defined as L-arabino-(4-O-methyl-D-glucurono)-D-xylan in sugarcane bagasse [14], the enzymatic hydrolysis of this biomass releases essentially glucose and xylose. *Saccharomyces cerevisiae*, the most used yeast in industrial 1G ethanol production, remains one of the leading candidates for 2G ethanol process due to its robustness and tolerance to industrial conditions. However, it does not naturally ferment xylose, and genetically modified strains often exhibit greater sensitivity to stresses at industry, compromising fermentation performance [15].

In this context, non-conventional yeasts (other than *S. cerevisiae*), specifically native ethanol producers capable of assimilating glucose, xylose, and other carbon sources. represent promising candidates for 2G ethanol production. For instance, *Kluyveromyces marxianus*, a thermotolerant yeast (35–45 °C), has demonstrated an efficient fermentation of glucose into ethanol using lignocellulosic hydrolysates [16]. *K marxianus* also can assimilate xylose for producing xylitol as main product [17]. Alternatively, *Scheffersomyces stipitis* is capable of fermenting both glucose and xylose into ethanol under appropriate conditions [18].

In this study, an integrated process for 2G ethanol production from sugarcane bagasse was developed by combining alkaline-sulfite-CTM pretreatment, enzyme loading optimization, and direct slurry fermentation along with a performance comparison of different yeast strains. The pretreatment was carried out with the aim of removing high content of lignin maintaining most of the cellulose and hemicellulose fractions. Then, the enzymatic hydrolysis of the pretreated material was evaluated using different loadings of commercial cellulase supplemented with β-glucosidase. Subsequently, the effects of temperature and nutrient supplementation on the fermentability of the obtained slurry were studied, employing *K. marxianus* Y-6860, *S. cerevisiae* PE-2, and *S. stipitis* Y-7124. Additionally, the slurry was supplemented with commercial glucose up to industrially relevant conditions for simulating the effect of high sugar levels on yeasts performance.

## Materials and methods

### Obtention and pretreatment of raw material

Sugarcane bagasse was supplied by a sugar and ethanol production plant from Sao Paulo region, BR. Firstly, this raw material was naturally dried until it reached approximately 12% moisture content and then stored for subsequent processing.

The alkaline-sulfite-CTM pretreatment was carried out in a two-step process, simulating industrial refining of CTM pulping. Firstly, 0.6 kg (dry mass) of sugarcane bagasse was impregnated with alkaline-sulfite liquor and cooked in a 16 L stainless-steel reactor, under previously defined conditions (10 g Na_2_SO_3_ + 5 g NaOH/100 g bagasse, 120 °C, 120 min, and solid-to-liquid ratio of 1:10) [13]. Afterwards, the obtained material (without washing) was resuspended in water to a final volume of 25 L (2.4% consistency) and refined in a Bauer MD-300 disk refiner (REGMED, Brazil) at conditions detailed by Mendes et al. [12]. The chemical composition of the raw and alkaline-sulfite-CTM-pretreated material was determined according to the Laboratory Analytical Procedure (LAP) of the National Renewable Energy Laboratory (NREL) [19].

### Enzyme preparations

Cellulase from *Trichoderma reesei* (Cellubrix) and β-glucosidase from *Aspergillus niger* (Novozyme 188) were used in this study (both from Novozymes Corp, DK). The filter paper activities of Cellubrix and Cellubrix + Novozyme 188 mixtures were determined according to the NREL-LAP [20], a procedure which in turn was based on the standard methodology of the International Union of Pure and Applied Chemistry [21]. β-glucosidase activity of Novozyme 188 was determined separately using *p*-nitrophenyl-β-D-glucopyranoside (*p*-NPG) as substrate, as described elsewhere [22].

### Enzymatic hydrolysis

The enzymatic hydrolysis of the alkaline-sulfite-CTM-pretreated material was evaluated using Cellubrix supplemented with Novozyme 188 at three different total enzyme loadings (9, 18, 27 FPU/g biomass), as described in Table 1. The enzymatic hydrolysis process was carried out in 50 mL Erlenmeyer flasks, containing 2.0 g (dry mass) of the pretreated material suspended in 50 mM sodium citrate buffer (pH 4.8) at 8% (w/v) solids loadings. The flasks were incubated in a thermostatic bath (MaxQ 7000, Thermo Scientific) at 45 °C and 100 rpm. Samples were withdrawn periodically, until 48 h of process, and immediately centrifuged at 3800 rpm x 20 min (Heraeus Megafuge 16R, Thermo Scientific). After centrifugation, the enzymes were inactivated by boiling for 5 min, and the supernatant was stored at 4 °C for further analysis of released sugars.

**Table 1:** Enzyme loadings for the enzymatic hydrolysis of alkaline-sulfite-CTM-pretreated sugarcane bagasse using cellulase (Cellubrix) supplemented with β-glucosidase (Novozyme 188)

Enzyme loadings	Cellubrix (FPU/g biomass)	Novozyme 188 (UI/g biomass)	Cellubrix + Novozyme 188 mixture (FPU/g biomass)
I	5	10	9
II	10	20	18
III	15	30	27

### Microorganisms and inoculum

*K. marxianus* Y-6860, *S. cerevisiae* PE-2, and *S. stipitis* Y-7124 were the yeast strains used for fermentation assays. These strains were maintained in malt extract agar slants at 4 °C until use. The inoculum preparation was carried out as described by Tomás-Pejó et al. [23].

### Evaluation of the slurry fermentability using experimental design

For fermentation assays, the slurry (liquid hydrolysate + residual solids, without solid-liquid separation) obtained by the enzymatic hydrolysis at (10 FPU Cellubrix + 20 UI Novozyme 188)/g biomass was used. The effect of nutrient supplementation and different conditions of temperature on the slurry fermentability was evaluated using a 2^2^ factorial design with center points, performed independently for each yeast strain (*K. marxianus* Y-6860, *S. cerevisiae* PE-2, and *S. stipitis* Y-7124).

These experiments were carried out in 125 mL Erlenmeyer flasks containing 48 mL of slurry supplemented or not with 2 mL of nutrient solution, according to the experimental conditions detailed in Table 3. In the assays without nutrient supplementation, distilled sterile water was added instead. Before inoculation, the pH of the fermentation media was adjusted to 5.0 using H_2_SO_4_ 1 M. Finally, the flasks were inoculated with 1 g L^− 1^ of cells and incubated in a shaker (NI 1712, Nova Instruments) at 150 rpm for 20 h. Periodic samples were collected and centrifuged (2000 rpm x 15 min) to monitor the fermentation process.

### Ethanol production at high-glucose concentration

After determining the most suitable conditions for fermenting the slurry, the effect of high-glucose concentration on ethanol production by the strains (*K. marxianus* Y-6860, *S. cerevisiae* PE-2, and *S. stipitis* Y-7124) was evaluated in replicate experiments. For this purpose, approximately 3.7 g of (≥ 99.5%) anhydrous glucose (Sigma-Aldrich, FR) was added to 50 mL of slurry in 125 mL Erlenmeyer flasks, while maintaining the nutrient concentrations (2.0 g L^− 1^ (NH_4_)_2_SO_4_, 1.0 g L^− 1^ KH_2_PO_4_, 0.3 g L^− 1^ MgSO_4_.7H_2_O, and 5.0 g L^− 1^ yeast extract). This way, a total glucose concentration of 100.0 g L^− 1^ was achieved in the fermentation medium, simulating the effect of high sugar levels on the performance of each yeast. The flasks containing the fermentation medium were inoculated with 1.0 g L^− 1^ of cells and incubated in shaker at 30 °C and 150 rpm. Samples were taken periodically and then centrifuged at 2000 rpm x 15 min to measure sugar consumption and ethanol production.

### Parameters calculations

The cellulose and xylan conversion yields during the enzymatic hydrolysis were calculated as specified by Silva et al. [24]. For fermentation assays, the ethanol yield factor ($${Y_{{\raise0.7ex\hbox{$P$} \!\mathord{\left/ {\vphantom {P S}}\right.\kern-0pt}\!\lower0.7ex\hbox{$S$}}}}$$, g g^− 1^) was calculated as the ratio of ethanol produced to substrate (glucose + xylose) consumed; and the ethanol volumetric productivity ($${Q_P}$$, g L^− 1^ h^− 1^) was defined as the ratio of maximum produced ethanol to the fermentation time. All kinetic profiles of both enzymatic hydrolysis and fermentation were analyzed using Origin^®^ software v2024 (OriginLab Corporation, Northampton, MA, USA), fitting the experimental curves.

### Analytical methods

Concentrations of sugars (glucose, xylose, and arabinose), acetic acid, xylitol, and ethanol were determined by High-Performance Liquid Chromatography (HPLC) (Agilent Technologies 1260 Infinity). A refractive index detector (RID) and a BIO-RAD Aminex HPX-87 H column (300 × 7.8 mm) were used at conditions previously detailed [24].

### Statistical analysis

The software Statistica^®^ v14.0 (TIBCO Software Inc., 2020, San Ramon, CA, USA) was used for statistical analysis. The magnitude of the effects on factors was determined by Pareto charts as well as by the analysis of variance (ANOVA). Considering the exploratory nature of the factorial design, and the inherent variability of biological systems, a 90% confidence level (α = 0.10) was adopted to identify potentially significant effects.

## Results and discussion

### Chemical composition of raw and pretreated material

Alkaline-sulfite-CTM pretreatment has proved to be an efficient strategy for enhancing the enzymatic hydrolysis of cellulose and hemicellulose, by reducing the inherent recalcitrance of lignocellulosic biomass [13]. The processing conditions of this pretreatment were previously established using sugarcane bagasse [12]. In the present study, the results of chemical composition analysis of sugarcane bagasse before and after alkaline-sulfite-CTM pretreatment are summarized in Table 2.

**Table 2:** Chemical composition of raw and alkaline-sulfite-CTM pretreated material

Components	Composition (% w/w)	
	Raw material	Alkaline-sulfite-CTM pretreated material
Glucan	38.9 ± 0.7	45.7 ± 0.9
Hemicellulose	24.2 ± 0.5	25.7 ± 0.6
Xylan	22.3 ± 0.4	23.4 ± 0.5
Arabinosyl	1.9 ± 0.1	2.3 ± 0.1
Acetyl groups	3.2 ± 0.5	0.12 ± 0.1
Total lignin	23.1 ± 0.2	15.1 ± 0.2
Acid insoluble lignin (AIL)	19.3 ± 0.2	10.6 ± 0.2
Acid soluble lignin (ASL)	3.8 ± 0.1	4.4 ± 0.1
Extractives	6.0 ± 0.7	5.5 ± 0.1
Ash	3.1 ± 0.7	8.3 ± 0.0

Total mass recovery after alkaline-sulfite-CTM pretreatment was 81.8%

Sugarcane bagasse is a lignocellulosic biomass composed of approximately 36 to 48% cellulose, 22 to 32% hemicellulose, 17 to 32% lignin, 2 to 14% extractives and 2% ash [25]. As shown in Table 2, alkaline-sulfite-CTM pretreatment triggered a significant reduction in total lignin content (47%) and a moderate reduction in extractives content (25%) in the raw material. Notably, the acetyl groups were almost completely (97%) removed using the alkaline-sulfite-CTM pretreatment. The removal of these components is critical since lignin acts as a physical barrier that limits enzyme access, while acetyl groups, bound to hemicellulose backbone, might inhibit productive binding between enzyme and polysaccharide in lignocellulose biomass [26]. Furthermore, during fermentation of lignocellulose biomass hydrolysates, acetyl groups are present in the form of acetic acid, which is an inhibitor of ethanol-producing yeasts. Therefore, deacetylation [27] and mechanical (disk) refining [28] of lignocellulose biomass are advantageous for both enzymatic hydrolysis and fermentation.

Due to the removal of above-mentioned components in different proportions, the relative content of polysaccharides (cellulose + hemicellulose) increased, totaling approximately 71.4% (w/w). Based on the mass balance of each component considering 81.8% total mass recovery after pretreatment, mass losses of cellulose – determined as glucan – and hemicellulose were only 4 and 13%, respectively. These results are comparable to those previously reported by Mendes et al. [12], which demonstrated that the resulting changes in chemical composition and structure increased fiber swelling and water retention capacity in sugarcane bagasse, thereby enhancing the enzymatic hydrolysis of cellulose and hemicellulose. Thus, the pretreated material obtained in the present study represents a suitable substrate for establishing a rational enzyme dosage for efficient enzymatic hydrolysis, as described in the following section.

### Effect of different enzyme loadings on enzymatic hydrolysis

**Fig. 1:**
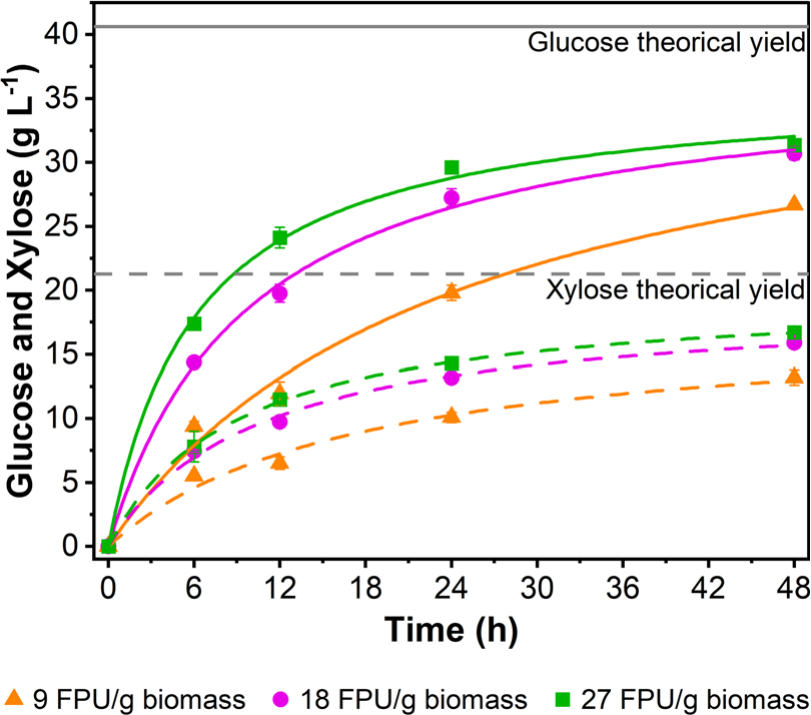
Enzymatic hydrolysis performance in terms of glucose (continuous lines) and xylose (dashed lines) release using different total enzyme loadings of Cellubrix supplemented with Novozyme 188

Figure 1 shows the performance of enzymatic hydrolysis of alkaline-sulfite-CTM pretreated material using three different total enzyme loadings (9, 18, and 27 FPU/g biomass) of Cellubrix supplemented with Novozyme 188, according to Table 1. In all assays, maximum glucose and xylose concentrations were reached after 48 h of reaction, with initial-fast rates (0–6 h) of enzymatic hydrolysis increasing as the enzyme loading increased. The subsequent decline in hydrolysis rates, transitioning to an intermediate (6–24 h) and slow (24–48 h) phases, can be attributed to the progressively increasing recalcitrance of the pretreated material [29, 30].

The lowest levels of hydrolysis were observed at the lowest enzyme loading (9 FPU/g of biomass), reaching cellulose and xylan conversion yields of 65 and 61%, respectively, after 48 h. Increasing the enzyme loading to 18 FPU/g biomass resulted in 31.0 g L^− 1^ of glucose and 15.7 g L^− 1^ of xylose after 48 h, corresponding to cellulose and xylan conversion yields of 76 and 74%, respectively. However, further increasing the enzyme loading to 27 FPU/g biomass showed only marginal improvements in terms of sugars concentrations, with cellulose and xylan conversion yields increasing by 3 and 6%, respectively, as compared to those obtained using 18 FPU/g biomass. This behavior has already been described by Silva et al. [24], who also evaluated the enzymatic supplementation of commercial enzyme cocktails – Cellic CTec2 and Viscozyme L – and observed that increasing the enzyme loadings beyond a certain limit does not significantly improve carbohydrate conversions yields. Additionally, it was considered that the potential for cellobiose to inhibit cellulases was limited – given the predominant β-glycosidase activity of Novozyme 188. Consequently, the obtained results may indicate limited enzyme accessibility to polysaccharides within the matrix of the alkaline-sulfite-CTM-pretreated material. Therefore, Cellubrix supplemented with Novozyme 188 loaded at a total 18 FPU/g biomass was considered the most suitable enzyme loading for the hydrolysis of alkaline-sulfite-CTM pretreated sugarcane bagasse. In addition, 70% cellulose conversion from lignocellulosic biomass has been suggested to be effective while using the lowest possible enzyme [30]. Thus, in the present work, an effective hydrolysis of both cellulose and xylan (approximately 75% conversion yield) was achieved by using commercial β-glycosidase supplementation of cellulase at a rational enzyme dosage, that was, using an enzyme loading that maximizes sugar release per unit of enzyme while maintaining high hydrolysis yield.

### Effects of temperature and nutrient supplementation on slurry fermentability employing different yeast strains

To produce sufficient slurry for the subsequent fermentation experiments, the enzymatic hydrolysis at the optimum enzyme loading was performed in replicate. The resulting slurry contained 2.41 ± 0.2 g L^− 1^ cellobiose, 27.19 ± 1.3 g L^− 1^ glucose, 14.85 ± 0.7 g L^− 1^ xylose, 13.03 ± 0.8 g L^− 1^ arabinose, and 0.50 ± 0.2 g L^− 1^ acetic acid. Then, the effects of the experimental variables temperature and nutrient supplementation of the slurry were simultaneously investigated using an experimental design as shown in Table 3. The results were expressed in terms of ethanol yield factor ($${Y_{{\raise0.7ex\hbox{$P$} \!\mathord{\left/ {\vphantom {P S}}\right.\kern-0pt}\!\lower0.7ex\hbox{$S$}}}}$$), ethanol volumetric productivity ($${Q_P}$$) and final product concentration. In addition, the ANOVA tables and regression coefficients for each response are presented in Tables S1–S6 of Supplementary Information.

**Table 3:** Experimental design and results according to the 2^2^ factorial design with center points, performed independently for each yeast strain, to assess the fermentability of the enzymatic slurry

Yeast strain	Variables^a^				Responses		Ethanol (g L^− 1^)
	Coded levels		Original levels^b^		$${Y_{{\raise0.7ex\hbox{$P$} \!\mathord{\left/ {\vphantom {P S}}\right.\kern-0pt}\!\lower0.7ex\hbox{$S$}}}}$$ (g g^− 1^)	$${Q_P}$$^c^ (g L^− 1^ h^− 1^)	
	*X*_1_	*X*_2_	*X*_3_	*X*_4_			
*K. marxianus* Y-6860	– 1	– 1	30	A	0.407	1.583	9.50
	1	– 1	40	A	0.451	2.067	12.40
	– 1	1	30	P	0.483	3.250	13.00
	1	1	40	P	0.411	3.163	12.65
	0	0	35	P/2	0.493	3.183	12.73
	0	0	35	P/2	0.493	3.208	12.83
	0	0	35	P/2	0.487	3.195	12.78
*S. cerevisiae* PE-2	– 1	– 1	30	A	0.453	0.958	5.75
	1	– 1	40	A	0.486	0.750	4.50
	– 1	1	30	P	0.472	2.174	13.04
	1	1	40	P	0.444	2.148	12.89
	0	0	35	P/2	0.359	1.827	10.96
	0	0	35	P/2	0.358	1.867	11.20
*S. stipitis* Y-7124	– 1	– 1	30	A	0.371	0.405	8.10
	1	– 1	40	A	0.271	0.125	2.50
	– 1	1	30	P	0.482	0.950	19.00
	1	1	40	P	0.271	0.405	8.10
	0	0	35	P/2	0.358	0.657	13.14
	0	0	35	P/2	0.380	0.685	13.70

^a^${X_1}$: temperature (°C); ${X_2}$: nutrient supplementation

^b^*A* absent, *P* present (2.0 g L^− 1^ (NH_4_)_2_SO_4_, 1.0 g L^− 1^ KH_2_PO_4_, 0.3 g L^− 1^ MgSO_4_.7H_2_O, and 5.0 g L^− 1^ yeast extract); P/2: concentration values by halves

^c^Values calculated considering the highest ethanol peak and the corresponding fermentation time: at 4 h for *K. marxianus* Y-6860, at 6 h for *S. cerevisiae* PE-2 and at 20 h for *S. stipitis* Y-7124

As can be seen in Table 3 for *K. marxianus* Y-6860, the $${Y_{{\raise0.7ex\hbox{$P$} \!\mathord{\left/ {\vphantom {P S}}\right.\kern-0pt}\!\lower0.7ex\hbox{$S$}}}}$$ values varied slightly (0.41–0.49 g g^− 1^) with the highest values observed under the center point conditions of temperature and nutrient supplementation. A similar trend was observed for the $${Q_P}$$ values, reaching up to 3.21 g L^− 1^ h^− 1^, which was higher than that (1.87 g L^− 1^ h^− 1^) observed for *S. cerevisiae* PE-2 under comparable conditions (30 °C with concentration of nutrients by halves). It is also noteworthy that *S. cerevisiae* PE-2 produced ethanol with a relatively high $${Q_P}$$ value (approximately 2.16 g L^− 1^ h^− 1^) in the presence of nutrients at 30 and 40 °C. Although the optimal fermentation temperature for *S. cerevisiae* is typically 30–35 °C [31], the intrinsic genetic diversity of the PE-2 strain confers desirable phenotypes, enabling it to thrive under harsh industrial environments [32, 33]. Besides that, neither *K. marxianus* nor *S. cerevisiae* showed significant xylose uptake (≤ 15%) under the evaluated conditions (data not shown). This behavior can be primarily explained by the absence of specific transporters and enzymes required for the natural assimilation of pentoses by *S. cerevisiae*, whereas *K. marxianus* possesses multiple transporters involved in pentose uptake [34]. Thus, the lack of co-utilization of glucose and xylose by *K. marxianus* is likely associated with glucose repression [35]. On the other hand, *S. stipitis* is an efficient pentose-metabolizing yeast [18]. In the present study, *S. stipitis* Y-7124 reach up to 90% of xylose consumption under the lowest condition of temperature (30 °C) with nutrient supplementation. These conditions resulted in a $${Y_{{\raise0.7ex\hbox{$P$} \!\mathord{\left/ {\vphantom {P S}}\right.\kern-0pt}\!\lower0.7ex\hbox{$S$}}}}$$ value of 0.48 g g^− 1^; however, the $${Q_P}$$ value (0.95 g L^− 1^ h^− 1^) was lower than those values achieved by *K. marxianus* and *S. cerevisiae*.

**Fig. 2:**
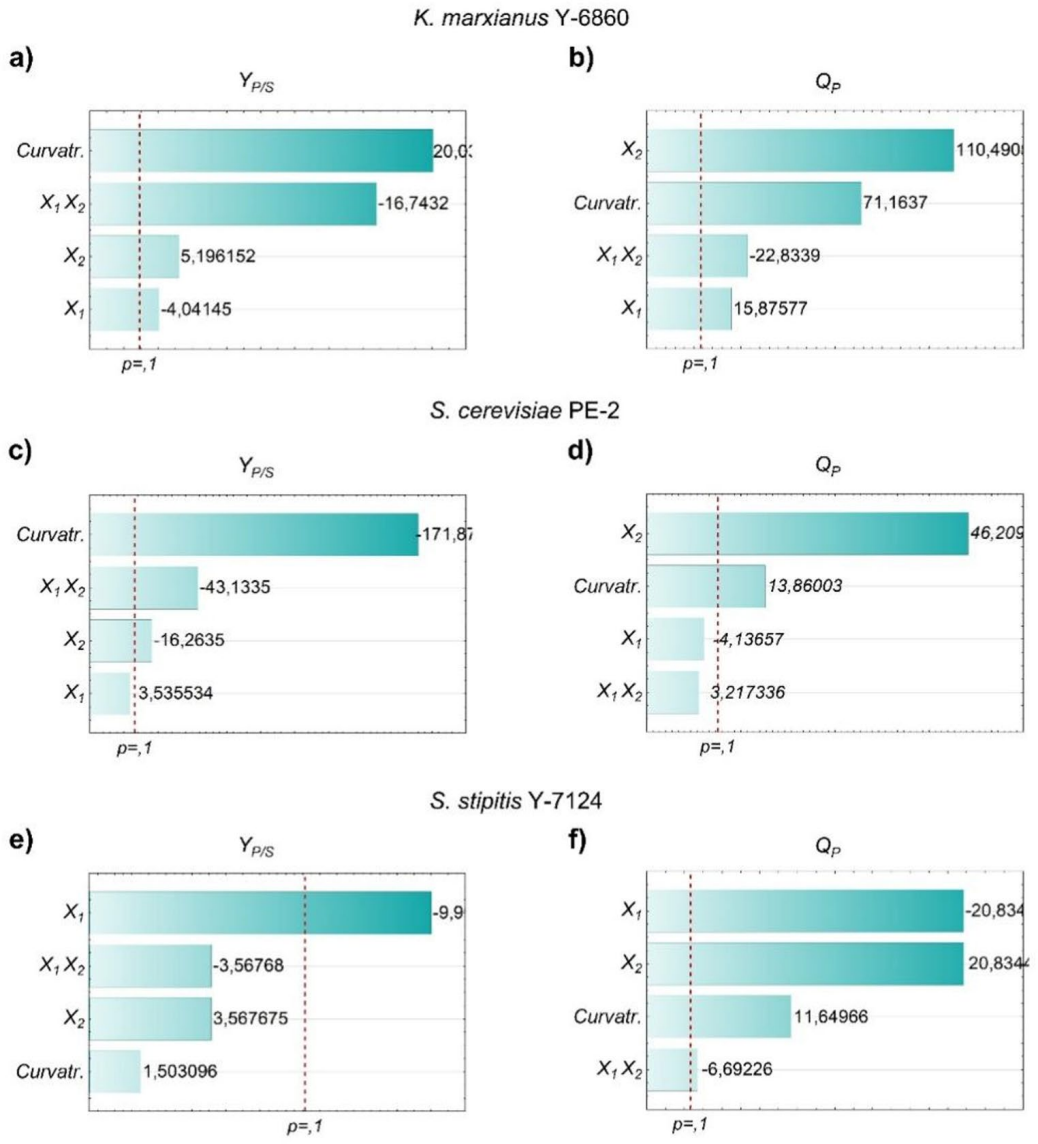
Pareto charts of the standardized effects of temperature ($${X_1}$$) and nutrient supplementation ($${X_2}$$) on the response variables $${Y_{{\raise0.7ex\hbox{$P$} \!\mathord{\left/ {\vphantom {P S}}\right.\kern-0pt}\!\lower0.7ex\hbox{$S$}}}}$$ (**a**, **c**, and **e**) and $${Q_P}$$ (**b**, **d**, and **f**) by *K. marxianus* Y-6860, *S. cerevisiae* PE-2, and *S. stipitis* Y-7124, respectively

In order to identify significant individual and interaction effects between the two factors on the evaluated fermentative parameters, a statistical analysis was performed independently for each yeast strain. The significance of the effects of temperature (${X_1}$), nutrient supplementation (${X_2}$), and interaction (${X_1}{X_2}$) on the response variables were evaluated using Pareto Charts of standardized effects at α = 0.1 as shown in Fig. 2.

Regarding the effects on $${Y_{{\raise0.7ex\hbox{$P$} \!\mathord{\left/ {\vphantom {P S}}\right.\kern-0pt}\!\lower0.7ex\hbox{$S$}}}}$$ by *K. marxianus* Y-6860 (Fig. 2a), curvature was the most significant effect (+ 20.0), confirming a non-linear response identified due to addition of center points in the design. The interaction term ($${X_1}{X_2}$$) was also significant, but with a negative effect (-16.7). Among the linear terms, $${X_2}$$ showed a positive and significant effect (+ 5.2), whereas $${X_1}$$ had a smaller and negative contribution (-4.0). These results suggest that increases in nutrient concentration enhance $${Y_{{\raise0.7ex\hbox{$P$} \!\mathord{\left/ {\vphantom {P S}}\right.\kern-0pt}\!\lower0.7ex\hbox{$S$}}}}$$, but this effect is modulated by temperature. Similar positive impacts of nutrient supplementation on *K. marxianus* performance have been reported previously [16]. For response $${Q_P}$$ (Fig. 2b), $${X_2}$$ term showed the strongest positive effect (+ 110.5), followed by curvature (+ 71.2), whereas $${X_1}{X_2}$$ term was negative (-22.8) and $${X_1}$$ term had a minor contribution. Overall, temperature was the least influential factor for both responses ($${Y_{{\raise0.7ex\hbox{$P$} \!\mathord{\left/ {\vphantom {P S}}\right.\kern-0pt}\!\lower0.7ex\hbox{$S$}}}}$$ and $${Q_P}$$), highlighting the thermotolerant nature of *K. marxianus*, which showed robust performance across the evaluated temperature range (30–40 °C).

Concerning effects on $${Y_{{\raise0.7ex\hbox{$P$} \!\mathord{\left/ {\vphantom {P S}}\right.\kern-0pt}\!\lower0.7ex\hbox{$S$}}}}$$ by *S. cerevisiae* PE-2 (Fig. 2c), curvature was the major effect (-171.9), indicating a strong non-linear response. The interaction term ($${X_1}{X_2}$$) also showed a significant negative effect (-43.1). On the other hand, response $${Q_P}$$ (Fig. 2d) was mainly impacted by $${X_2}$$ term with a positive effect (+ 46.2), followed by curvature (+ 13.9). It indicated that ethanol productivity increased as nutrient supplementation increased, showing a moderate curvature across the evaluated conditions. It is worth noting that temperature had a limited influence on both responses ($${Y_{{\raise0.7ex\hbox{$P$} \!\mathord{\left/ {\vphantom {P S}}\right.\kern-0pt}\!\lower0.7ex\hbox{$S$}}}}$$ and $${Q_P}$$), consistent with the known tolerance of the PE-2 strain to industrial conditions, such as elevated temperatures and oxidative stress [32].

For response $${Y_{{\raise0.7ex\hbox{$P$} \!\mathord{\left/ {\vphantom {P S}}\right.\kern-0pt}\!\lower0.7ex\hbox{$S$}}}}$$ by *S. stipitis* Y-7124 (Fig. 2e), $${X_1}$$ term was the only significant factor, with a negative effect (− 10.0), indicating reduced ethanol yield as temperature increased. A similar negative effect of $${X_1}$$ (-20.8) was also observed on response $${Q_P}$$ (Fig. 2f), whereas $${X_2}$$ term (+ 20.8) and curvature (+ 11.6) showed significant positive effects. These results indicated that fermentation performance of *S. stipitis* Y-7124 was favored at lower temperatures and higher nutrient concentrations within the evaluated range. This agrees with Campos et al. [36], who reported decreased ethanol production by *S. stipitis* NRRL 7124 as temperature increases from 32 to 35 °C. Therefore, the results obtained in the present study suggest that ethanol fermentative performance of *S. stipitis* Y-7124 is highly temperature-sensitive. However, the observed effects cannot be attributed exclusively to temperature, since oxygen transfer was not directly controlled in the experiments performed in shaking flasks. Therefore, this response may be partly associated with reduced O_2_ solubility at higher temperatures, which may further limit oxygen availability. Although *S. stipitis* performs best under microaerobic conditions for ethanol production, excessively low oxygen availability can impair xylose metabolism by affecting cofactor regeneration and redox balance, ultimately reducing ethanol [37].

**Fig. 3:**
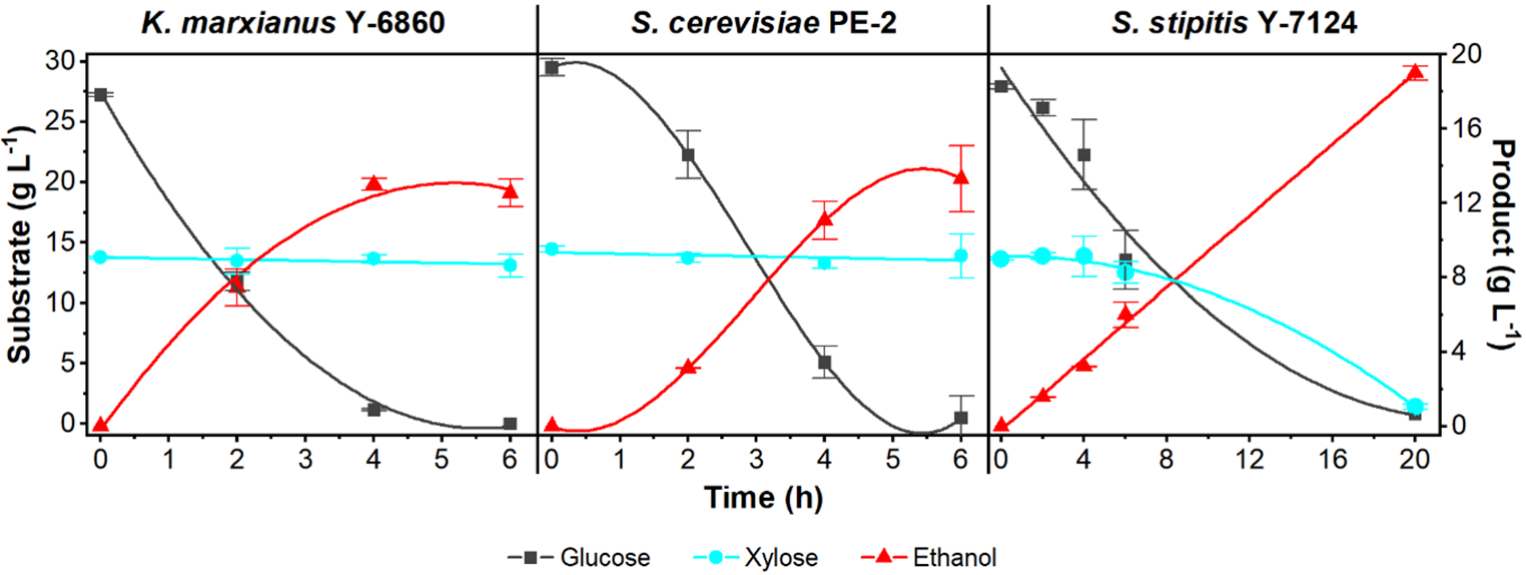
Fermentation profile of *K. marxianus* Y-6860, *S. cerevisiae* PE-2, and *S. stipitis* Y-7124 at 30 °C in the enzymatic slurry supplemented with nutrients (2.0 g L^− 1^ (NH_4_)_2_SO_4_, 1.0 g L^− 1^ KH_2_PO_4_, 0.3 g L^− 1^ MgSO_4_.7H_2_O, and 5.0 g L^− 1^ yeast extract)

As above-mentioned, the $${Y_{{\raise0.7ex\hbox{$P$} \!\mathord{\left/ {\vphantom {P S}}\right.\kern-0pt}\!\lower0.7ex\hbox{$S$}}}}$$ response of *S. stipitis* Y-7124 could be adequately described by a first-order model including only the main effect of temperature; whereas the significance of the curvature observed for the responses of *K. marxianus* Y-6860 and *S. cerevisiae* PE-2 indicated some deviation from linearity, suggesting that second-order effects could improve model fitting. However, the experimental region already covered conditions that yield ethanol ($${Y_{{\raise0.7ex\hbox{$P$} \!\mathord{\left/ {\vphantom {P S}}\right.\kern-0pt}\!\lower0.7ex\hbox{$S$}}}}$$ ≥ 0.47 g g^− 1^) very close to the theoretical maximum ($${Y_{{\raise0.7ex\hbox{$P$} \!\mathord{\left/ {\vphantom {P S}}\right.\kern-0pt}\!\lower0.7ex\hbox{$S$}}}}max$$ = 0.51 g g^− 1^). Therefore, further expansion of the design with axial or face-centered points was not pursued, as it was unlikely to yield substantial gains in process optimization. Consequently, the optimal conditions for the slurry fermentation, employing *K. marxianus* Y-6860, *S. cerevisiae* PE-2, and *S. stipitis* Y-7124 were established as temperature of 30 °C with nutrient supplementation consisting of 2.0 g L^− 1^ (NH_4_)_2_SO_4_, 1.0 g L^− 1^ KH_2_PO_4_, 0.3 g L^− 1^ MgSO_4_.7H_2_O, and 5.0 g L^− 1^ yeast extract. The corresponding fermentation profiles obtained from the factorial design experiments are shown in Fig. 3.

Regarding substrate consumption at optimal conditions (Fig. 3), both *K. marxianus* Y-6860 and *S. cerevisiae* PE-2 completely consumed glucose after 5 h of fermentation, with a relatively higher consumption rate observed for *K. marxianus*. This highlights the potential of *K. marxianus* when compared to the well-established industrial strain of *S. cerevisiae*. In contrast, *S. stipitis* Y-7124 required approximately 20 h to fully consume the glucose. Despite its slower glucose uptake, *S. stipitis* Y-7124 was the only strain capable of substantially consuming xylose (~ 90%) and performing co-fermentation [38], which occurred after glucose levels had decreased to ~ 12 g L^− 1^. After 20 h of fermentation, only about 3% of the total sugars remained in the enzymatic slurry.

In terms of ethanol production (Fig. 3), *K. marxianus* Y-6860 and *S. cerevisiae* PE-2 reached similar concentrations (~ 13 g L^− 1^) after 5 h, reflecting efficient glucose fermentation. Although *S. stipitis* Y-7124 required a longer fermentation time (~ 20 h), it produced a higher concentration of ethanol (~ 19 g L^− 1^), attributed to xylose conversion. These results demonstrate that *K. marxianus* is highly efficient in the rapid conversion of glucose to ethanol, with performance comparable to that of *S. cerevisiae*, while *S. stipitis* stands out for its ability to convert both C6 and C5 sugars into ethanol.

It is important to note in Fig. 3 that all assays showed complete glucose exhaustion and, in one of them, nearly complete xylose uptake by *S. stipitis*. It indicates the feasibility of carrying out fermentation of the enzymatic slurry, derived from the alkaline-sulfite-CTM pretreated sugarcane bagasse, without a prior solid-liquid separation step, which is advantageous for integrating process into 2G ethanol production [16] and reducing energy consumption [39].

### Effect of high-glucose concentration on ethanol production

One key challenge for the technical and economic viability of 2G ethanol production is achieving industrial relevant ethanol concentrations (≥ 50 g L^− 1^) [40], which in turn requires initial sugar concentrations of at least 100 g L^− 1^. In this context, robust microorganisms are needed which can tolerate process-related stresses, such as high osmotic pressure resulting from elevated sugar concentrations and product inhibition by ethanol.

**Fig. 4:**
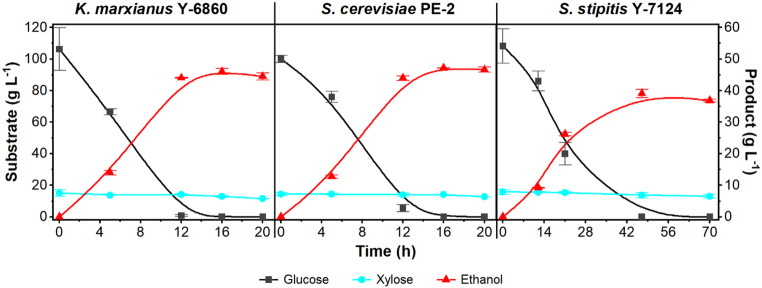
Fermentation profile of *K. marxianus* Y-6860, *S. cerevisiae* PE-2, and *S. stipitis* Y-7124 at 30 °C in the enzymatic slurry supplemented with nutrients (2.0 g L^− 1^ (NH_4_)_2_SO_4_, 1.0 g L^− 1^ KH_2_PO_4_, 0.3 g L^− 1^ MgSO_4_.7H_2_O, and 5.0 g L^− 1^ yeast extract) and containing 100 g L^− 1^ glucose

To evaluate the fermentative capacity of *K. marxianus* Y-6860, *S. cerevisiae* PE-2, and *S. stipitis* Y-7124, the enzymatic slurry was supplemented with glucose to a final concentration of 100 g L^− 1^, and fermentations were conducted under optimal conditions evaluated (30 °C with nutrient supplementation). The corresponding fermentation profiles are shown in Fig. 4, and the calculated fermentative parameters are summarized in Table 4.

**Table 4:** Fermentative parameters of ethanol production at high-glucose concentration in the enzymatic slurry at 30 °C supplemented with nutrients, employing different yeast strains

Parameters	Yeast strains		
	K. marxianus Y-6860	S. cerevisiae PE-2	S. stipitis Y-7124
$${Y_{{\raise0.7ex\hbox{$P$} \!\mathord{\left/ {\vphantom {P S}}\right.\kern-0pt}\!\lower0.7ex\hbox{$S$}}}}$$ (g g^− 1^)	0.41 ± 0.04	0.47 ± 0.01	0.36 ± 0.02
${Q_P}$ (g L^− 1^ h^− 1^)	2.82 ± 0.13^a^	2.95 ± 0.00^a^	0.70 ± 0.02^a^
${Q_{Glu}}$ (g L^− 1^ h^− 1^)	6.79 ± 0.62^a^	6.25 ± 0.14^a^	1.93 ± 0.20^a^
${Q_{Xyl}}$ (g L^− 1^ h^− 1^)	0.14 ± 0.09^a^	0.04 ± 0.03^a^	0.03 ± 0.01^a^
*η * (%)	80.1 ± 4.5	92.0 ± 2.4	70.0 ± 4.2

^a^Values calculated considering the highest ethanol peak and the corresponding fermentation time: at 16 h (*K. marxianus* Y-6860 and *S. cerevisiae* PE-2) and at 56 h (*S. stipitis* Y-7124)

Regarding substrate consumption (Fig. 4), *K. marxianus* Y-6860 and *S. cerevisiae* PE-2 completely consumed glucose within 16 h of fermentation, whereas *S. stipitis* Y-7124 required 56 h. These times also corresponded to the maximum ethanol concentrations, reaching 46.0, 47.1, and 38.6 g L^− 1^ for *K. marxianus*, *S. cerevisiae*, and *S. stipitis*, respectively. Consequently, ethanol volumetric productivity from glucose (${Q_{Glu}}$) for *K. marxianus* and *S. cerevisiae* were approximately 3.5-times higher than those observed for *S. stipitis* (Table 4).

Specifically, the fermentation parameters obtained for *K. marxianus* Y-6860 (Table 4) were comparable to those ($${Y_{{\raise0.7ex\hbox{$P$} \!\mathord{\left/ {\vphantom {P S}}\right.\kern-0pt}\!\lower0.7ex\hbox{$S$}}}}$$ = 0.43 g g^− 1^; $${Q_P}$$ = 1.02 g L^−1^ h^−1^; *η * = 84.8%) previously reported by Ferreira et al. [16] using an enzymatic slurry, derived from deacetylated rice straw, supplemented with nutrients. These authors reported better performance ($${Y_{{\raise0.7ex\hbox{$P$} \!\mathord{\left/ {\vphantom {P S}}\right.\kern-0pt}\!\lower0.7ex\hbox{$S$}}}}$$ = 0.46 g g^−1^; $${Q_P}$$ = 1.74 g L^−1^ h^−1^; *η * = 90.2%) when residual solids were removed prior to fermentation; however, the $${Q_P}$$ value remained lower than that (Table 4) obtained in the present study. It is also important to mention that fermentation experiments, employing *K. marxianus*, were performed using enzymatic slurries derived from the alkaline-sulfite-CTM-pretreated biomass, both washed and unwashed, to evaluate the potential effect of retained salts. The two conditions showed similar fermentation performance in terms of $${Y_{{\raise0.7ex\hbox{$P$} \!\mathord{\left/ {\vphantom {P S}}\right.\kern-0pt}\!\lower0.7ex\hbox{$S$}}}}$$, $${Q_P}$$, *η *, and final ethanol concentration (data not shown). Therefore, this suggests that the enzymatic slurry derived from the alkaline-sulfite-CTM-pretreated sugarcane bagasse provided favorable conditions for fermentation without the need for a solid-liquid separation step. Additionally, it was demonstrated the robust fermentative performance of *K. marxianus* Y-6860 under high-glucose conditions.

For *S. cerevisiae* PE-2, the fermentation performance observed in this study (Table 4) was also higher than the values of ${Q_P}$ = 1.94 g L^−1^ h^−1^ and *η * = 87.3% reported elsewhere [41] for the same industrial strain during fermentation of an enzymatic hydrolysate, derived from acid-alkali pretreated sugarcane bagasse, supplemented with nutrients (yeast extract and KH₂PO₄). These differences reinforce the influence of biomass pretreatment on slurry fermentability, likely due to differences in inhibitor profiles generated during acid pretreatment, including furans, acetic acid and phenolic compounds [42].

The fermentation parameters obtained (Table 4) for *S. stipitis* Y-7124 were comparable to those ($${Y_{{\raise0.7ex\hbox{$P$} \!\mathord{\left/ {\vphantom {P S}}\right.\kern-0pt}\!\lower0.7ex\hbox{$S$}}}}$$ = 0.39 g g^− 1^; $${Q_P}$$ = 0.45 g L^−1^ h^−1^) reported by Okonkwo et al. [43], who observed incomplete utilization of sugars at high concentrations, with glucose being preferentially consumed over xylose. Unlike in previous experiments (Fig. 3), no xylose uptake was detected under conditions of high-glucose concentration (Fig. 4). This suggests strong catabolite repression under the evaluated glucose:xylose levels, likely due to transport competition with glucose [44]. As a result, fermentation of C5 mixed with C6 sugars requires extended periods due to sequential utilization of mixed sugars, negatively affecting $${Q_P}$$. Furthermore, operational parameters such as aeration and agitation may have affected the distribution of metabolic flux between biomass formation and ethanol production [45]. Therefore, better control of oxygen transfer and mixing conditions may increase $${Y_{{\raise0.7ex\hbox{$P$} \!\mathord{\left/ {\vphantom {P S}}\right.\kern-0pt}\!\lower0.7ex\hbox{$S$}}}}$$ and favor xylose fermentation into ethanol by *S. stipitis*.

The present study essentially provides a comparative assessment of conventional and non-conventional yeasts for direct fermentation of alkaline-sulfite-CTM-derived enzymatic slurry without solid-liquid separation using different yeast strains under both moderate and high-sugar concentrations relevant to industrial 2G ethanol production. Supplementation of the slurry to approximately 100 g L⁻¹ total sugars improved ethanol production, yielding a maximum ethanol concentration of 47.1 g L⁻¹. Although this value remains slightly below the ethanol titer (50 g L⁻¹) considered desirable for the techno-economic feasibility of industrial 2G ethanol production, titers above 40 g L⁻¹ have also been recognized as economically relevant and represent a substantial improvement in process performance [46]. Therefore, the results demonstrate the potential of the proposed integrated process while also indicating that further optimization is required.

Ethanol production from fermentation media containing mixed C6/C5 sugars is also influenced by the sugar balance, and the glucose-to-xylose ratio can vary considerably depending on the lignocellulosic feedstock and processing conditions. Despite the promising fermentation performance observed, especially for *K. marxianus*, glucose repression may have limited the efficient co-utilization of C6 and C5 sugars by *S. stipitis* under conditions of high sugar concentration, highlighting the importance of optimizing sugar composition and fermentation conditions for the conversion of mixed sugars. In this sense, beyond adaptative laboratory evolution and metabolic engineering strategies [46], approaches such as fed-batch fermentation and the co-cultivation of *S. cerevisiae* or *K. marxianus* with *S. stipitis* could be explored to alleviate glucose repression and improve the co-utilization of C6 and C5 sugars at high-glucose concentrations, thereby increasing ethanol titers [47–50]. Future studies will focus on further exploring these strategies to promote the viability of 2G ethanol production.

## Conclusion

The alkaline-sulfite-CTM pretreatment of sugarcane bagasse produced a solid material with minimal loss of carbohydrates. This pretreated material was efficiently hydrolyzed, resulting in a sugar-rich slurry, through the β-glucosidase supplementation of cellulase, at a rational enzyme dosage. Then, optimal conditions of temperature and nutrient supplementation were established for the slurry fermentation, employing three different yeast strains. *K. marxianus* Y-6860 exhibited high ethanol production potential with fermentative performance comparable to that of *S. cerevisiae* PE-2 in terms of $${Y_{{\raise0.7ex\hbox{$P$} \!\mathord{\left/ {\vphantom {P S}}\right.\kern-0pt}\!\lower0.7ex\hbox{$S$}}}}$$ and $${Q_P}$$, rapidly exhausting glucose for ethanol production. In contrast, *S. stipitis* Y-7124 required longer fermentation times but demonstrated its capacity to consume both glucose and xylose for ethanol production. When the slurry was supplemented up to 100 g L^− 1^ glucose, *K. marxianus* Y-6860 and *S. cerevisiae* PE-2 maintained robust fermentation performance, while *S. stipitis* Y-7124 exhibited limited xylose uptake during the evaluated period, with carbon catabolic repression being a possible cause. Overall, the results demonstrate the potential of the obtained slurry for direct fermentation without the need for solid-liquid separation, which may improve process integration and efficiency in 2G ethanol production.

## Supplementary Information

Below is the link to the electronic supplementary material.

Supplementary Material 1

## Data Availability

No datasets were generated or analysed during the current study.
