# Supplementary material for: Comparative fermentability of a glucose/xylose enriched enzymatic slurry using different yeast strains
Source: Bioprocess Biosyst Eng. 2026 Jul 24;49(8):2303–15. doi: 10.1007/s00449-026-03399-3 (PMC13424243; doi:10.1007/s00449-026-03399-3)
Supplement: Supplementary file 1 — Supplementary Material 1 [file 449_2026_3399_MOESM1_ESM.pdf]

Supplementary Information

Comparative fermentability of a glucose/xylose enriched enzymatic slurry using different yeast strains

Lina M. Durán [a]; Miguel A. D. Flores-Alarcón [a]; Adriane M. F. Milagres [a]; Inês C. Roberto [a,*]

[a] Department of Biotechnology, Engineering College of Lorena, University of São Paulo (USP), Estrada Municipal do Campinho, N° 100, Campinho, Lorena, SP 12602-810, Brazil.

*Corresponding author email address: iroberto@usp.br

Table S1 Analysis of variance (ANOVA) of the main and interaction effects of temperature (X_1) and nutrient supplementation (X_2) on the $Y_{P/S}$ of *K. marxianus* Y-6860

Source	Sum of Squares	df	Mean Square	F-value	p-value
Model	0.0039	3	0.0013	107.89	0.0092
A-Temperature	0.0002	1	0.0002	16.33	0.0561
B-Nutrient supplementation	0.0003	1	0.0003	27.00	0.0351
AB	0.0034	1	0.0034	280.33	0.0035
Curvature	0.0048	1	0.0048	401.29	0.0025
Pure Error	0.0000	2	0.0000		
Cor Total	0.0087	6			
R^2 = 0.99			Adjusted-R^2 = 0.98		

Table S2 Analysis of variance (ANOVA) of the main and interaction effects of temperature (X_1) and nutrient supplementation (X_2) on the Q_P of *K. marxianus* Y-6860

Source	Sum of Squares	df	Mean Square	F-value	p-value
Model	2.03	3	0.6765	4327.20	0.0002
A-Temperature	0.0394	1	0.0394	252.04	0.0039
B-Nutrient supplementation	1.91	1	1.91	12208.16	<0.0001
AB	0.0815	1	0.0815	521.39	0.0019
Curvature	0.7917	1	0.7917	5064.27	0.0002
Pure Error	0.0003	2	0.0002		
Cor Total	2.82	6			
R^2 = 0.99			Adjusted-R^2 = 0.99		

Table S3 Analysis of variance (ANOVA) of the main and interaction effects of temperature (X_1) and nutrient supplementation (X_2) on the $Y_{P/S}$ of *S. cerevisiae* PE-2

Source	Sum of Squares	df	Mean Square	F-value	p-value
Model	0.0011	3	0.0004	712.50	0.0275
A-Temperature	6.250E-06	1	6.250E-06	12.50	0.1755
B-Nutrient supplementation	0.0001	1	0.0001	264.50	0.0391
AB	0.0009	1	0.0009	1860.50	0.0148
Curvature	0.0148	1	0.0148	29540.17	0.0037
Pure Error	5.000E-07	1	5.000E-07		
Cor Total	0.0158	5			
R^2 = 0.99			Adjusted-R^2 = 0.99		

Table S4 Analysis of variance (ANOVA) of the main and interaction effects of temperature (X_1) and nutrient supplementation (X_2) on the Q_P of *S. cerevisiae* PE-2

Source	Sum of Squares	df	Mean Square	F-value	p-value
Model	1.73	3	0.5767	720.92	0.0274
A-Temperature	0.0137	1	0.0137	17.11	0.1510
B-Nutrient supplementation	1.71	1	1.71	2135.31	0.0138
AB	0.0083	1	0.0083	10.35	0.1918
Curvature	0.1537	1	0.1537	192.10	0.0459
Pure Error	0.0008	1	0.0008		
Cor Total	1.88	5			
$R^2 = 0.99$			Adjusted-$R^2 = 0.99$		

Table S5 Analysis of variance (ANOVA) of the main and interaction effects of temperature (X_1) and nutrient supplementation (X_2) on the $Y_{P/S}$ of *S. stipitis* Y-7124

Source	Sum of Squares	df	Mean Square	F-value	p-value
Model	0.0303	3	0.0101	41.79	0.1131
A-Temperature	0.0242	1	0.0242	99.92	0.0635
B-Nutrient supplementation	0.0031	1	0.0031	12.73	0.1740
AB	0.0031	1	0.0031	12.73	0.1740
Curvature	0.0005	1	0.0005	2.26	0.3737
Pure Error	0.0002	1	0.0002		
Cor Total	0.0311	5			
$R^2 = 0.99$			Adjusted-$R^2 = 0.97$		

Table S6 Analysis of variance (ANOVA) of the main and interaction effects of temperature (X_1) and nutrient supplementation (X_2) on the Q_P of *S. stipitis* Y-7124

Source	Sum of Squares	df	Mean Square	F-value	p-value
Model	0.3579	3	0.1193	304.31	0.0421
A-Temperature	0.1702	1	0.1702	434.07	0.0305
B-Nutrient supplementation	0.1702	1	0.1702	434.07	0.0305
AB	0.0176	1	0.0176	44.79	0.0944
Curvature	0.0532	1	0.0532	135.71	0.0545
Pure Error	0.0004	1	0.0004		
Cor Total	0.4115	5			
$R^2 = 0.99$			Adjusted-$R^2 = 0.99$		
